# Cortisol Awakening Response, Internalizing Symptoms, and Life Satisfaction in Emerging Adults

**DOI:** 10.3390/ijms18122501

**Published:** 2017-11-27

**Authors:** Li Shen Chong, Michelle Thai, Kathryn R. Cullen, Kelvin O. Lim, Bonnie Klimes-Dougan

**Affiliations:** 1 Department of Psychology, College of Liberal Arts, University of Minnesota, Minneapolis, MN 55455, USA; chong051@umn.edu (L.S.C.); thaix049@umn.edu (M.T.); 2 Department of Psychiatry, School of Medicine, University of Minnesota, Minneapolis, MN 55454, USA; rega0026@umn.edu (K.R.C.); kolim@umn.edu (K.O.L.)

**Keywords:** hypothalamic pituitary adrenal axis, hypothalamic pituitary adrenal (HPA), cortisol, cortisol awakening response (CAR), emerging adults, risk, life satisfaction

## Abstract

The cortisol awakening response (CAR) has been associated with depression and a broader range of internalizing problems. Emerging adulthood is characterized by numerous stressful transitional life events. Furthermore, the functioning of the neurobiological stress system changes across development. These considerations underscore the importance of evaluating the physiological stress system in emerging adults in identifying the extent to which cortisol levels vary with risk and protective factors for mental health. The present study evaluated the association between internalizing symptoms and perceived life satisfaction with CAR in 32 young adults. Three saliva samples were collected to measure cortisol levels upon awakening and participants completed the Depression Anxiety Stress Scale (DASS) and Satisfaction with Life Scale (SWLS). Results show a significant positive correlation between area under the curve for CAR with internalizing symptoms (DASS total) and the DASS-depression subscale, but not with life satisfaction. Study limitations, implications, and future directions for these finding were discussed.

## 1. Introduction

The transition between adolescence and adulthood, commonly referred to as emerging adulthood, is often protracted in highly industrialized nations. In the period lasting from age 18 years to the mid-twenties, emerging adults are often in the process of gaining the education and training needed prior to joining the workforce, establishing a residence, and starting a career [1]. Emerging adulthood, particularly for those attending college, is a stage of life that may also include exploring their identities, feeling unstable or “in between”, focusing on themselves, and experiencing a wide range of relational possibilities, such as selecting a mate [1]. As emerging adults experience life events associated with stressful inter- and intrapersonal problems, there is a surge in the incidence of psychopathology during this developmental period [2].

Critical biological systems are developing when emerging adults are facing these life transitions. Specifically, attunement of the stress activation and regulation systems takes place during this time period, along with the physiological stress system, which includes the hypothalamic pituitary adrenal (HPA) axis [3]. Cortisol, one of the key stress hormones regulated by the HPA axis, is associated with stress, somatic illness, and psychological disorders [4]. Although the detrimental effects of negative psychological states or traits on physical and mental health have become increasingly recognized, the functioning and clinical relevance of these systems during the specific developmental period of emerging adulthood remain poorly characterized.

In the area of stress neurobiology research, recent attention has focused on the cortisol awakening response (CAR). CAR involves the natural response of the HPA axis to awakening. Typically, a healthy individual experiences a sharp increase in cortisol secretion in the morning after waking, and then cortisol levels decline throughout the day. Specifically, there is an increase in cortisol levels within first 15 to 45 min following awakening [5]. The function of the CAR is yet unknown, but it is likely a part of the awakening process that is associated with rapid reciprocal switching between subcortical and cortical brain regions [6] as well as the physiological reactivity changes observed in response to stress [7]. This pattern of CAR is evident from childhood through adulthood, but with subtle changes noted across development [5].

Individual differences in HPA axis functioning might provide risk markers for emotional disorders [8,9]. Elevation of the CAR is associated with major depression [10,11] and possibly with anxiety disorders [12,13]. One of the largest CAR studies conducted to date by Vreeburg and colleagues [14] showed that adults (mean age: 44 years) with a diagnosis of a depressive or an anxiety disorder had an elevated CAR. Additionally, there is evidence that HPA axis dysregulation, including elevated CAR, might represent not only a biomarker for psychological disorders, but also a trait vulnerability to certain psychological disorders [11,14]. However, the findings have been more mixed when considering a dimensional approach to characterizing risk. For example, studies have found high neuroticism or harm avoidance to be positively [15,16], negatively [17], or not [11] associated with CAR. Research shows that psychosocial factors may be related to the discrepancy in CAR’s directionality. Measures of job stress and general life stress were positively correlated with CAR, whereas fatigue, burnout, and exhaustion were negatively correlated with CAR [18]. Some of the mixed findings when considering dimensions of risk may be due to the fact that most studies with adults address a wide age group and do not typically account for development (for some exceptions refer to [19,20]).

While risk has been systematically assessed with regard to CAR, protective processes have rarely been studied. Evidence suggests that protective factors such as positive affect and optimism are associated with low cortisol levels [21]. Since life satisfaction, the general affective evaluation of a person’s life [22], has been shown to be a reliable predictor of good health outcomes [23], it may serve as a protective factor. However, there has been less of an emphasis of how life satisfaction may influence physiological processes. We are only aware of one study to date that attempted to assess the links between life satisfaction and CAR [24]. They found a trend showing that low life satisfaction was associated with lower CAR among adults between the ages 35 and 84 years [24].

The purpose of this study was to examine the association between CAR and risk and protective factors of psychopathology in emerging adulthood using a dimensional approach. We predicted that high levels of internalizing symptoms (problems with stress, depression, and anxiety) would serve as a risk factor for stress system dysregulation and would be associated with elevated CAR. Second, we predicted that life satisfaction would serve as a protective factor, minimizing the impact of negative states and mitigating cortisol dysregulation. Third, we predicted that life satisfaction may moderate the impact of internalizing symptoms of CAR.

## 2. Results

This study involved 32 (age 18–27 years; 17 females) college students recruited from the University of Minnesota. Table 1 provides descriptive data of participant characteristics, indexes of psychological functioning, and salivary cortisol measures. All DASS and SWLS scores were within the normal range. Therefore, elevations on the DASS in this sample represent risk markers as opposed to reflections of current psychopathology. The mean cortisol levels at three different time points after awakening showed the expected diurnal variation: a rise in morning cortisol levels.

As shown in Table 2, the summary scores for DASS (Depression, Anxiety, Stress, and Total) were moderately to highly correlated (*r*’*s* = 0.45–0.89). The DASS and the SWLS were inversely related (*r*’*s* = −0.23 to −0.35), although the findings did not reach significance. As hypothesized, the DASS-total and DASS-depression subscales were significantly positively correlated with AUCi CAR. These findings held when we conducted follow-up analyses using partial correlations controlling for age, sex, and time of awakening. However, the relationship between DASS-anxiety and DASS-stress and AUCi CAR were not significant. There were also no significant correlations between any of the DASS scales and AUCg. There were no significant correlations between SWLS and either the AUCg or AUCi.

Moderation analyses revealed that the interaction between SWLS and, respectively, with DASS-depression and DASS-total was non-significant (Table 3).

## 3. Discussion

The primary aim of the study was to identify the extent to which cortisol levels vary with mental health risk and protective factors (internalizing symptoms and perceived life satisfaction) among emerging adults. Consistent with predictions, internalizing symptoms were positively associated with cortisol levels within the first half-hour of awakening. The depressive symptom subscale was driving the association between internalizing problems and CAR. While there is also tentative evidence from past research showing a link between anxiety and stress and CAR [25,26], our results failed to confirm this finding. It is possible that this study failed to find evidence of this link because symptoms of anxiety and stress are more widely distributed across the population. Another consideration is that due to our small sample size (discussed further below), we were under-powered to detect a weaker relationship with stress and anxiety symptoms. A third possibility is that while most previous studies examine how trait measures relate to CAR, this study uses DASS as a measure of state depression and anxiety.

The current study adds to the literature by considering a dimensional approach to evaluating internalizing symptoms, suggesting that those experiencing depressive symptoms may be particularly more vulnerable to HPA axis dysregulation. The results of this study underscore the importance of how we define and measure the pattern of salivary cortisol at awakening. The two indexes of cortisol summary scores used here represent somewhat different aspects of CAR. AUCg represents an index of overall cortisol elevation. Other studies have mostly used similar indexes of cortisol at awakening to show a relation with depression and other internalizing symptoms (e.g., anxiety and panic). While the results of this study showed a positive correlation for AUCg and depressive symptoms, this relationship was only at a trend (*r* = 0.33, *p* = 0.06). AUCi is another index characterizing CAR, which more accurately captures the elevation of subsequent samples relative to the first sample of cortisol. There is only a small pool of studies that have reported on this index. Consistent with our findings, previous research has shown that elevations in AUCi were found among currently depressed and remitted participants, but not healthy subjects [13]. A positive association with hopelessness reactivity was also shown with AUCi [27]. On the other hand, Powell and Scholtz [25] suggested that elevated AUCi helped to reduce distress responses to daily life stress. Although an elevated AUCi may indicate an increase in the availability of resources to address stress, the findings of our study show that this index was linked with symptoms of risk.

Given that life satisfaction and health outcomes are interlinked, it would be reasonable to predict that higher life satisfaction can act as a protective factor against CAR-related issues. Contrary to predictions, there was no systematic relationship noted for protective factors and CAR. Considering that internalizing symptoms and life satisfaction are not bipolar constructs, these constructs may, in fact, be largely independent factors [28]. Also, life satisfaction was not a moderator for the correlation between internalizing symptoms and CAR. The results may have been limited because participants were generally satisfied with their lives (e.g., between the “slightly agree” to “agree” rating), as the pool of young adults were mastering critical developmental tasks (e.g., taking steps to prepare for their vocation by attending college).

There are several limitations that should be taken into account when drawing conclusions from this study. First, the sample size was small, raising the risk both for under-detection of important relationships between CAR and anxiety/stress and also for the possibility of spurious results. Replication with a larger sample size is needed to establish with certainty the association between risk and protective factors and CAR. Second, different evaluation tools may vary in their scope in assessing risk and protective processes. For example, perceived stress in Powell and Schlotz’s [25] study primarily stems from work overload, whereas the DASS that was used in this study includes a more subjective self-established definition of stress. While the DASS is considered an index of state functioning, there is evidence that ratings on DASS are also related to more enduring features of psychopathology [27,29]. Third, careful training was provided to participants to complete these home salivary collections, but there are several improvements that should be made to future research on this topic. The window used to assess CAR in this study was based on the 30-min period in which a rise in cortisol is expected. However, ideally, more samples should be collected during an extended awakening window (60 min), so that the evaluation of both the typical rise and fall in cortisol levels could be measured. While all participants provided the samples, the date/time was written in by participants; future research using objective time stamps will be important to address this limitation. A related issue that has not been sufficiently addressed, neither in this study nor past research, is how to confirm the precise time of awakening to establish accurate estimates of CAR. However, if collection biases had been present, they would have likely obscured our results rather than enhanced them [30]. The current study examined CAR from a single day. Assessments over at least two days is recommended to increase reliability of this index [31], as previous research has suggested that the CAR of a single day is more reflective of state rather than trait measure [32]. Finally, the external validity of this study may be limited because all the participants were college students, who do not fully represent emerging adulthood [33]. Participants in this study were further limited to those who were interested in learning meditative techniques to regulate their stress, rather than specifically targeting highly stressed subgroups of young adults, such as first-generation college students [2].

## 4. Method

### 4.1. Participants and Procedures

This study was approved by the University of Minnesota Institutional Review Board, #1201M09222 (approved 20.02.2012). A screening phone interview was conducted in an effort to recruit participants who were either free from or had less severe forms of mental illness. Then, participants met with research staff to complete the consent process, a diagnostic interview using Mini International Neuropsychiatric Interview (MINI) [34], and self-report scales including the Depression Anxiety Stress Scales (DASS) and Satisfaction with Life Scale (SWLS). Participants were given instructions for how to provide salivary samples, which were obtained within a week of the initial assessment. Although the current work focuses on the baseline assessments of clinical and HPA axis functioning, participants in this study were initially recruited by flyers with the possibility of participating in a randomized control meditation intervention trial. A subset of the participants went on to participate in the intervention trial; the longitudinal data is not examined here.

### 4.2. Materials

Dimensional assessments of internalizing symptoms were assessed using the short version (21-item) of the Depression Anxiety Stress Scales (DASS) [29]. The DASS is a self-report questionnaire that uses a four-point Likert scale to assess the frequency and severity of three negative emotional states, common in internalizing problems (anxiety, depression, and stress), in the past week [35]. This measure has documented evidence of reliability and validity (e.g., [27,29]).

Protective factors were assessed by using the Satisfaction with Life Scale (SWLS), a five item, seven-point Likert-format self-report scale that accesses the overall subjective well-being of an individual [36]. This commonly used scale has a wealth evidence for acceptable reliability and validity (e.g., [36,37]).

CAR was based on three samples of salivary cortisol. Participants were given instructions and vials for salivary cortisol collection in their home during a “typical” weekday. They were then trained to fill three quarters of the vial (about 1 teaspoon or 5 mL) with saliva after chewing and spitting out Trident Original gum for 20–30 s, provide notations of date/time, etc., store their samples in their home freezer, and return the samples during their next visit. Participants were given instruction to collect their own saliva samples in three consecutive time points: at awakening, 15 min, and 30 min after waking. Samples were labeled and stored in a −25 °C freezer until they were shipped to Universität Trier in Trier, Germany for analysis. Researchers used assay methods consistent with standards [38]. Summary indexes of CAR included area under the curve with respect to ground (AUCg) and area under the curve with respect to increase (AUCi) [39].

### 4.3. Statistical Analyses

The primary aim of this study was to evaluate the relationship between risk and protective factors (negative states, life satisfaction) and CAR. We used Pearson correlations to evaluate these relationships. We also considered whether life satisfaction may moderate the effect of internalizing problems on the CAR. Cortisol values were winsorized so that outliers were within three standard deviations of the mean. Moderation analyses were conducted in SPSS using the PROCESS macro (refer to [40] for more details about the analysis).

## 5. Conclusions

In conclusion, the measurement of CAR can be a useful biomarker for assessing the vulnerability of those experiencing and internalizing depressive symptoms in the emerging adult population. Considering the developmental challenges faced by emerging adults, these results have implications for those navigating this developmental period. Targeted psychosocial interventions (e.g., [41,42]) that are aimed at reducing cortisol levels can incorporate these findings to effectively decrease the risk of CAR-related physical and mental health issues among emerging adults.

## Figures and Tables

**Table 1:** *Sample Characteristics* (*N* = 32).

Variables (*N* = 32)	Parameter	Range
Demographics		
Age—mean years, (SD)	20.47 (2.27)	18–27
Sex (female)	17 (53.1)	
Sampling factors		
Reported time of awakening—mean time, (SD)	8:04 am	6:20 am–10:30 am
Time window for salivary sample collection	31 min 45 s	29 min 0 s–56 min 59 s
Current Axis I diagnosis (MINI)—*n* (%)	4 (12.5%)	
DASS 21—mean (SD)		
Total	0.47 (0.39)	0.05–1.71
Depression	0.34 (0.41)	0.00–1.71
Anxiety	0.42 (0.49)	0.00–1.71
Stress	0.65 (0.50)	0.00–2.14
SWLS—mean (SD)		
Total	5.28 (1.02)	3.00–6.80
Cortisol indicators—mean (SD)		
CAR_1_, at awakening (μg/dL)	0.32 (0.20)	0.03–1.01
CAR_2_, +15 min (μg/dL)	0.53 (0.26)	0.04–1.13
CAR_3_, +30 min (μg/dL)	0.53 (0.31)	0.11–1.24
AUCg (μg/dL)	15.101 (7.84)	3.11–38.87
AUCi (μg/dL)	4.88 (5.35)	5.12–19.63

DASS = Depression, Anxiety, and Stress Scale, SWLS = Subjective Wellbeing of Life Scale, CAR = cortisol awakening response, AUCg = area under the curve with respect to ground, AUCi = area under the curve with respect to increase, MINI = Mini International Neuropsychiatric Interview.

**Table 2:** Bivariate correlation between covariates, AUC, SWLS, DASS scores.

Variable	1	2	3	4	5	6	7	8	9	10
1. Age										
2. Sex	0.03									
3. Reported time of awakening	−0.33	0.00								
4. Current axis I diagnosis (MINI)	0.05	0.36 *	0.06							
5. DASS total	−0.09	0.03	−0.03	−0.13						
6. DASS depression	0.00	0.00	−0.13	−0.16	0.83 **					
7. DASS anxiety	−0.25	0.02	−0.02	−0.02	0.82 **	0.45 **				
8. DASS stress	0.04	0.04	0.05	−0.17	0.89 **	0.69 **	0.57 **			
9. SWLS	−0.14	0.12	0.17	0.24	−0.35	−0.34	−0.23	−0.34		
10. AUCg	0.29	−0.04	−0.30	−0.08	0.24	0.33	0.11	0.22	0.01	
11. AUCi	−0.04	−0.01	−0.14	0.07	0.40 *	0.50 **	0.32	0.23	−0.17	0.55 *

** *p* ≤ 0.01; * *p* ≤ 0.05; Sex (male = 1, female = 2), DASS = Depression, Anxiety, and Stress Scale, SWLS = Subjective Wellbeing of Life Scale, CAR = cortisol awakening response, AUCg = area under the curve in respect to ground, AUCi = area under the curve in respect to increase. Results between AUCi and DASS (total and depression) remain significant when controlling for age, sex, or reported time of awakening.

**Table 3:** Interaction between AUCi, DASS total, DASS depression and SWLS.

**(A) AUCi Predicted from Interaction between DASS Depression and SWLS**				
d	B	SE	*t*	*p*
Constant	5.30 (3.29, 7.31)	0.98	5.41	0.00
SWLS total	−0.05 (−1.97, 1.87)	0.93	−0.05	0.96
DASS depression	6.23 (1.34, 11.12)	2.38	2.61	0.01
DASS depression × SWLS total	2.70 (−3.62, 9.01)	3.08	0.88	0.39
**(B) AUCi Predicted from Interaction between DASS Total and SWLS**				
	B	SE	*t*	*p*
Constant	5.20 (3.08, 7.30)	1.03	5.06	0.00
SWLS total	−0.25 (−2.30, 1.80)	1.00	−0.25	0.80
DASS total	5.01 (−0.21, 10.22)	2.54	1.97	0.06
DASS total × SWLS total	1.95 (−4.32, 8.21)	3.05	0.64	0.53
**(C) AUCi Predicted from DASS Depression and SWLS**				
	B	SE	*t*	*p*
Constant	4.85	0.88	5.52	0.00
SWLS total	0.01	0.93	0.01	1.00
DASS depression	6.62	2.33	2.84	0.008
**(D) AUCi Predicted from DASS Total and SWLS**				
	B	SE	*t*	*p*
Constant	4.92	0.93	5.31	0.00
SWLS total	−0.18	0.98	−0.18	0.86
DASS total	5.23	2.49	2.10	0.045

(**A**) *R*^2^ = 0.27; (**B**) *R*^2^ = 0.17; (**C**) *R*^2^ = 0.25; (**D**) *R*^2^ = 0.16.
